# CD86 is linked to apoptosis in canine histiocytic sarcoma

**DOI:** 10.3389/fvets.2025.1546047

**Published:** 2025-04-09

**Authors:** Benjamin Diehl, Andreas Kirchhoff, Florian Hansmann

**Affiliations:** ^1^Faculty of Veterinary Medicine, Institute of Veterinary Pathology, Leipzig University, Leipzig, Germany; ^2^Practice of Veterinary Pathology, Gelsenkirchen, Germany

**Keywords:** CD80, CD86, dog, histiocytic sarcoma, immune checkpoint, mx1, survivin

## Abstract

Immune checkpoints are critical for the regulation of tumor growth and regression. Recently an effect of CD80 and CD86 on tumor regression in canine cutaneous histiocytoma has been described. Further, the expression of MX dynamin like GTPase 1 (mx1) in cancer is linked to immune evasion. Thus, the present study aimed to investigate the effects of CD80 and CD86 in histiocytic sarcoma (HS), a rare and progressive malignancy in dogs and to elucidate the status of the interferon-I pathway. Twenty-two tissue samples of HS from skin, lung and liver of 15 dogs were used. Immunohistochemistry targeting CD80, CD86, programmed death-ligand 1 (PD-L1), survivin, cleaved caspase-3 (Casp-3), stimulator of interferon genes (STING) and mx1 was performed. Slides were digitized and analyzed with QuPath. The numbers of CD86- and Casp-3 expressing cells showed a positive correlation. In the skin and lung, numbers of CD80 immunolabeled cells were higher than for CD86, while CD80 and CD86 levels were comparable in the liver. In general, low numbers of PD-L1 immunolabeled tumor cells were detected. Intranuclear survivin expression was linked to Casp-3. Mx1 and STING were expressed in tumor cells. A possible link between CD86 and Casp-3 points to a role of CD86 in tumor cell death. The findings indicate relevant differences in CD80 and CD86 expression between organs and a function in histiocytic disease in dogs. Further, the expression of markers of the interferon-type-I pathway indicates a role in immune evasion.

## 1 Introduction

Histiocytes can be subdivided into dendritic cells (DC) or macrophages (1). Interstitial DC are part of the conventional DC and reside in non-lymphoid tissue such as skin and lung (1). They renew from bone marrow-derived progenitor cells. Langerhans cells (LC) on the other hand are a subset of specialized DC only occurring in the epidermis (1). They are similar in function to DCs, are formed during fetal development and are self-sustaining, but differ in their origin. Both subtypes have antigen presenting capacities and migrate to the tributary lymph nodes upon antigen sensing, where they recruit T-cells via costimulatory molecules such as CD80 and CD86 (1).

Histiocytic sarcoma (HS) is a malignant tumor, which derives from interstitial DC (2). Although rare in most breeds, Bernese Mountain Dogs, Flat Coated Retrievers, Rottweilers, and Golden Retrievers show a predisposition (1–3). A localized and a disseminated form of HS are described. The localized form most commonly arises in the skin, while the disseminated form shows lesions in spleen, lung, bone marrow, liver and lymph nodes (2). Disseminated HS has been discussed to be a progressive form of localized HS, but its exact pathogenesis remains unclear (2). In a study investigating the clinical outcome, dogs with localized HS survived significantly longer than those with disseminated HS while the breed had no impact on the survival (4). HS must be differentiated from other histiocytic diseases such as histiocytosis, canine cutaneous histiocytoma (CCH), LC histiocytosis and hemophagocytic histiocytic sarcoma in dogs. Histiocytosis occurs in a cutaneous and a disseminated form and is proposed to originate from interstitial DC as well. In contrast to HS, histiocytosis appears to be an immune-mediated disease (5, 6). Histologically, a perivascular pattern with a pleomorphic infiltrate and a tendency for vasodestructive growth is seen in histiocytosis (6). LC disorders occur rather frequently in dogs (3, 7). CCH originating from LC, is the most frequent benign histiocytic neoplasm in dogs (3, 7). The progressive LC histiocytosis is far less common and can result in death if systemic progression occurs (1). Another type of histiocytic disease in canines, named for its histologically prevalent feature of erythrophagocytosis, is hemophagocytic histiocytic sarcoma, which is derived from macrophages (8).

Immune checkpoints play a crucial role in the regulation of the immune response. Their functions often include T-cell inhibition and apoptosis (9). CD80 and CD86 belong to the group of immune checkpoint molecules and can, apart from their stimulatory function, convey immunomodulatory signals by binding to cytotoxic T-lymphocyte associated protein 4 (CTLA-4) on T cells (9). In the unaffected skin of dogs, interstitial DC express CD80 but not CD86 (10). It was recently demonstrated that in CCH, tumor cells express both markers (11, 12) and that a decrease in CD80 may have an impact on regression (11). In cancer, immune checkpoint molecules are often overexpressed and modulate the tumor microenvironment (TME) enabling tumor growth (9). Previous studies investigated the presence of immune checkpoint molecules and their receptors in canine tumors, including CCH, melanocytic neoplasia, mammary tumors and squamous cell carcinoma (11, 13–15). Conversely, in a case report of HS in a Great Dane CD86 was expressed while CD80 was not expressed (2, 16, 17).

Programmed death-ligand 1 (PD-L1) and its receptor programmed cell death protein 1 (PD-1) are immune checkpoint molecules, which have been linked to suppressed antitumor immunity in canine histiocytic sarcoma and lymphoma (18, 19). An evaluation of the therapeutic potential of monoclonal antibodies against PD-L1/PD-1 in dogs revealed no clear result (20), while some successes in humans have been described (21).

Another way neoplasia modulate the TME is via the interferon (IFN)-I pathway (22, 23). Stimulator of interferon genes (STING) is a mediator of IFN-I and plays an important role in cancer immunity (24). However, studies also show an immunosuppressive effect when the IFN-I response is constantly induced (24, 25). In brief, a chronic stimulation by IFN-I is responsible for the so called IFN-related DNA damage resistance signature (26). This signature includes IFN-stimulated genes, such as MX dynamin like GTPase 1 (mx1) (23, 26). In viral infection this serves as a mechanism against viral inhibition of the IFN-stimulated response by using unphosphorylated interferon stimulated gene factor 3 (ISGF3) for signaling (26). In human cancer this pattern is discussed to protect certain malignancies from radiotherapy (22) and mx1 has been linked with a poor prognosis in breast and prostate cancer as well as colorectal carcinoma in humans (27–29). Recently, in CCH an expression of mx1 has been described and a relation to the active immunological response is suggested (11). It remains unclear whether this is a specific phenomenon in CCH or whether the IFN-signaling pathway is a potential global target for influencing tumorigenesis.

The main hypotheses in the present study were (1) the immune checkpoint molecules CD80 and CD86 are linked to the progressive behavior of HS with possible organ specific differences and (2) the expression of mx1 is a result of an activated IFN-I pathway and can be linked to immune evasion.

## 2 Materials and methods

### 2.1 Characterization of samples

For this study formalin fixed paraffin embedded (FFPE) samples of 15 individual dogs with HS were used. Samples included 10 excisional skin biopsies and HS obtained from necropsy cases (6 HS in the lung and 5 HS in the liver; Table 1). Lung and liver samples were matched, if possible. The diagnosis in each case was confirmed by a board-certified veterinary pathologist (FH) using hematoxylin and eosin stain (HE) as well as immunohistochemistry targeting ionized calcium-binding adapter molecule 1 (Iba-1) and CD204.

**Table 1:** Summary of the patients included in the study.

Case number	Breed	Sex	Age (years)	Samples provided	Sampling method
1	Bernese Mountain Dog	Female	8	Skin	Excisional biopsy
2	Bernese Mountain Dog	Female	8	Skin, lung, liver	Necropsy
3	Bernese Mountain Dog	Male	9	Skin	Excisional biopsy
4	Bernese Mountain Dog	Female	9	Skin	Excisional biopsy
5	Bernese Mountain Dog	Male	6	Skin	Excisional biopsy
6	Beagle	Male	12	Skin	Excisional biopsy
7	Podenco-mix	Female	6	Skin	Excisional biopsy
8	Labrador	Male	10	Skin	Excisional biopsy
9	Golden Retriever	Male	10	Skin	Excisional biopsy
10	Flat Coated Retriever	Female	7	Skin	Excisional biopsy
11	Bernese Mountain Dog	Female	6	Lung, liver	Necropsy
12	German Shepherd Dog	Female	3	Lung, liver	Necropsy
13	Bernese Mountain Dog	Female	9	Lung, liver	Necropsy
14	Bernese Mountain Dog	Female	7	Lung, liver	Necropsy
15	Bernese Mountain Dog	Male	6	Lung	Necropsy

Mitotic count in tumor cells of the skin samples was evaluated by counting mitotic figures in 10 consecutive areas of 0.237 mm^2^ (2.37 mm^2^ in total) at 400-fold magnification using an Olympus BX53 microscope (Evident Europe GmbH, Hamburg, Germany).

### 2.2 Immunohistochemistry

Immunohistochemistry was performed as previously described (11). Briefly, slides were cut and deparaffinized. This was followed by heat-based epitope retrieval and blocking steps for endogenous peroxidase. Primary antibodies were incubated overnight at 4°C and the secondary antibody was incubated for 30 min at room temperature. Incubation with the avidin-biotin-complex (ABC; Vector Laboratories, Newark, CA, United States) was followed by chromogen incubation with 3,3′-diaminobenzidine (DAB; Sigma-Aldrich, St. Louis, MO, United States) and counterstaining with Mayer’s hematoxylin was used for visualization. Antibodies, their respective concentration, the method used for epitope retrieval and controls are given in Table 2. Furthermore, the subcellular localization of survivin immunostaining was evaluated as being “intranuclear” or “intracytoplasmic” in 100 cells per slide and a percentage was calculated.

**Table 2:** Antibodies used in the study.

Epitope	Control tissue	Supplier/product number	Working concentration	Epitope retrieval	Host/clonality	Secondary antibody	Supplier/product number	Detection system
Cleaved Caspase-3	Tonsil	R&D systems AF835	0.2 μg/mL	Citrate	Rabbit/polyclonal	Goat-anti-rabbit	Vector Laboratories BA-1000	ABC
CD80	Tonsil	Bioss Bs-10340R	1.25 μg/mL	Citrate	Rabbit/polyclonal	Goat-anti-rabbit	Vector Laboratories BA-1000	ABC
CD86	Tonsil	Antibodies-online ABIN736701	2.5 μg/mL	Citrate	Rabbit/polyclonal	Goat-anti-rabbit	Vector Laboratories BA-1000	ABC
CD204	Histiocytic sarcoma	Trans genic Inc. SRA-E5	0.5 μg/mL	Citrate	Mouse/monoclonal	Goat-anti-mouse	Vector Laboratories BA-9200	ABC
Iba-1	Tonsil	Novus bio NBP2-19019	0.06 μg/mL	Citrate	Rabbit/polyclonal	Goat-anti-rabbit	Vector Laboratories BA-1000	ABC
Ki-67	Small intestine	Dako MIB-1 M7240	0.92 μg/mL	Citrate	Mouse/monoclonal	Goat-anti-mouse	Vector Laboratories BA-9200	ABC
Mx1	Brain (canine distemper infected)	M143 (provided by Prof. Kochs, Institute of Virology, Freiburg University)	0.6 μg/mL	Citrate	Mouse/monoclonal	Goat-anti-mouse	Vector Laboratories BA-9200	ABC
PD-L1	Tonsil	Abcam 233482	0.5 μg/mL	EDTA	Rabbit/polyclonal	Goat-anti-rabbit	Vector Laboratories BA-1000	ABC
STING	Bronchus	Novus bio NBP2-24683	5 μg/mL	Citrate	Rabbit/polyclonal	Goat-anti-rabbit	Vector Laboratories BA-1000	ABC
Survivin	Squamous cell carcinoma	Novus bio NB500-201	5 μg/mL	Citrate	Rabbit/polyclonal	Goat-anti-rabbit	Vector Laboratories BA-1000	ABC

CD, cluster of differentiation; Iba-1, ionized calcium-binding adapter molecule 1; PD-L1, programmed death ligand 1; EDTA, ethylenediaminetetraacetic acid; STING, stimulator of interferon genes; ABC, avidin-biotin-complex.

### 2.3 Quantitative analysis using QuPath

Analysis followed the principle established before (11). Briefly, stained slides were scanned with the Zeiss Axioscan 7 (Zeiss Group, Jena, Germany) at 20× magnification. The whole-slide images were then evaluated in QuPath [vers. 5.0 (30)]. Processing included color deconvolution and choosing 10 tiles of 500 × 500 μm for evaluation. After cell detection, manually chosen intensity thresholds were applied to identify and calculate the percentage of immunopositive cells.

### 2.4 *In-situ* hybridization and analysis of transcript location

Representative areas of the different skin sample paraffin blocks were cut with a 3 mm biopsy punch and assigned to a new FFPE block. The expression of mx1 was then validated by detection of mRNA transcripts. This was achieved by using the RNAscope technology (Advanced Cell Diagnostics, Inc., Minneapolis, MI, United States) according to the manufacturer’s instructions. Probes were designed for mx1 (Entrez Gene ID 403745) and inflamed brain tissue from a canine distemper encephalitis case was used as a positive control. Process controls were used as described previously (11). The evaluation consisted of multiple steps: positive pixels for each core were detected with a pixel-based classifier and the percentage was calculated (“positive pixel”) and a median calculated for each core. The “positive cell” measurement was achieved by filtering cells with at least 1% positive pixels. The percentage of positive pixels per cell is described as “positive signal” and represented as median value per core.

### 2.5 Statistical analysis

Statistical analysis was done using R 4.4.1 with the RStudio and the *tidyverse* package (31–33). All measurements were checked for normal distribution using Q–Q-plots, after which medians and quantiles were calculated. Statistical significance was evaluated using the Kruskal–Wallis-test and Wilcoxon-rank-sum-test for *post hoc* analysis. A *p*-value <0.05 was chosen as threshold for statistical significance. Possible correlation was analyzed using Spearman’s *ρ*.

To facilitate an evaluation of the influence of CD80 and CD86, a ratio was calculated for each individual sample by dividing the respective median percentage for CD80 and CD86 positive cells. This ratio was then used to show possible correlation with Casp-3 and Ki-67 percentages. Moreover, the ratio of Ki-67 and Casp-3 positive cells was calculated in a similar manner and evaluated for correlation with tumor markers. Lastly, the available median positive proportions of CCH from our previous study were used to calculate similar ratios for CCH to enable a comparison of these with (sub) cutaneous HS.

## 3 Results

### 3.1 General characterization of the samples

Neoplastic cells in all samples were diffusely positive for Iba-1 and CD204 and showed an absence of vasotropism, vasodesctructive growth as well as epitheliotropism in the skin. Samples were characterized by minor to moderate infiltration of lymphocytes. Except for one specimen, all HS were nodular to multinodular and 12 out of 15 had multinucleated giant cells.

The samples originated from a total of 15 dogs with 6 male and 9 female animals. Nine of the investigated dogs were Bernese Mountain Dogs. Mitotic figures in 2.37 mm^2^ were 15.2 on average with a range of 3 to 47. Animals were on average 7.7 years old.

### 3.2 Markers for cell proliferation and apoptosis

**Figure 1:**
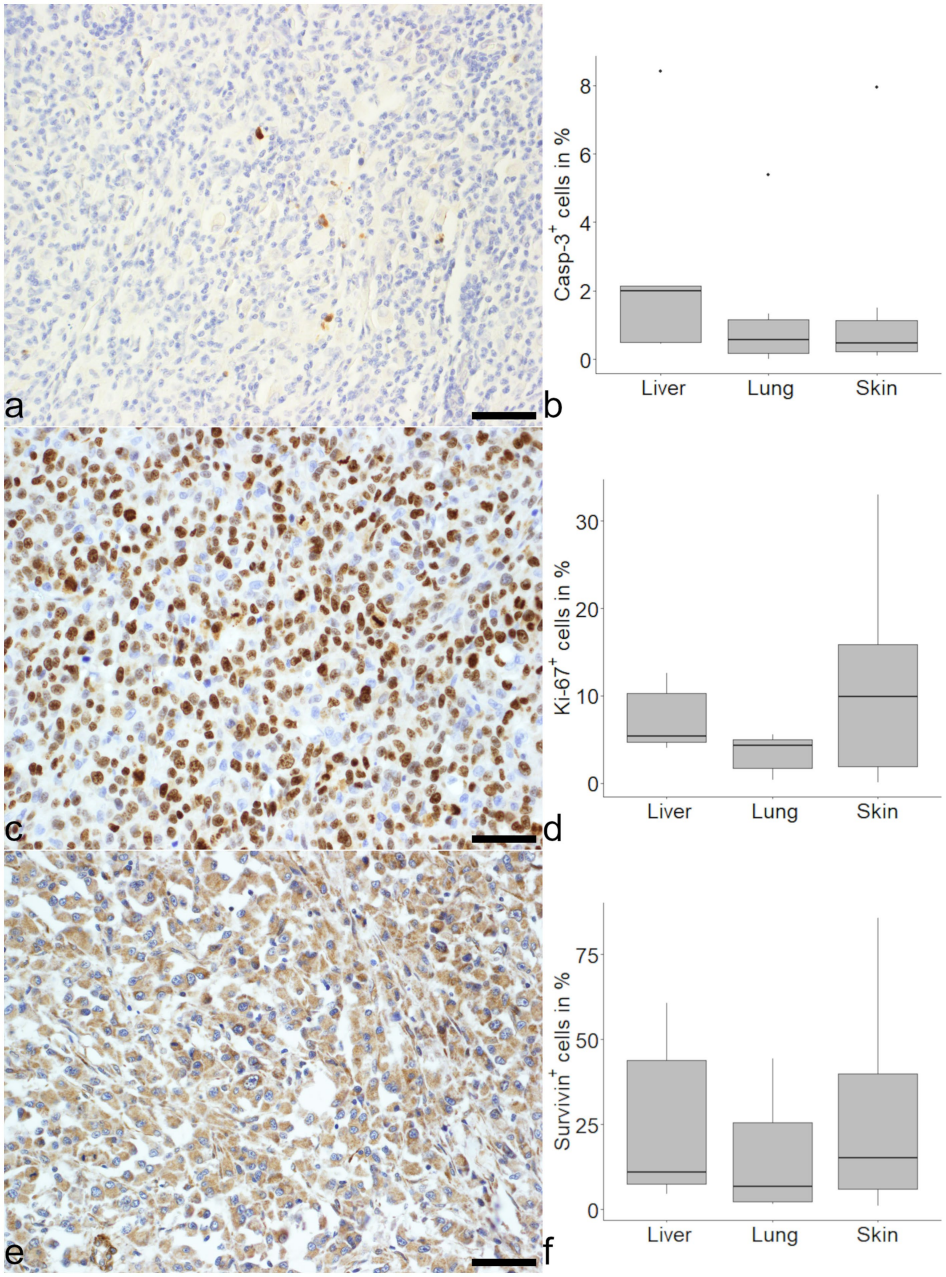
Evaluation of markers related to mitosis and apoptosis in histiocytic sarcoma (HS). An expression of cleaved caspase-3 (Casp-3) was seen in tumor cells of HS in the skin **(a)** and medians were similar in liver, lung and skin **(b)**. There were variably high numbers of Ki-67 positive cells in (sub) cutaneous HS **(c)** with no significant difference between the organs **(d)**. Survivin was expressed in varying amounts of tumor cells **(e)** and comparable between organs **(f)**. Mayer’s hematoxylin counterstain, bar = 50 μm.

Casp-3 immunohistochemistry showed median proportions of 2.00, 0.58, and 0.46% positive cells in liver, lung and skin, respectively (Figure 1). For Ki-67 median proportions of 5.34, 4.30, and 9.85% positive cells in liver, lung and skin were found (Figure 1). Both markers showed no significant differences between organs.

Survivin expression was notably heterogeneous. Proportions of 10.84% positive cells were found in the liver, 6.68% in the lung and 15.07% in the skin (Figure 1). In the majority of cells evaluated, the signal for survivin was localized in the cytoplasm. Considering the different organs, tumor cells in the skin showed a higher median proportion of intranuclear survivin with 19% as opposed to 3% in liver and 2% in the lung. The intranuclear localization of survivin was positively correlated with Ki-67 (*ρ* = 0.46, *p* < 0.05), but not with Casp-3 (*ρ* = 0.42, *p* = 0.18). In contrast, overall survivin expression in all organs was neither correlated with Ki-67 (*ρ* = 0.02, *p* = 0.33) nor Casp-3 (*ρ* = 0.12, *p* = 0.58).

### 3.3 Immune checkpoint molecules

**Figure 2:**
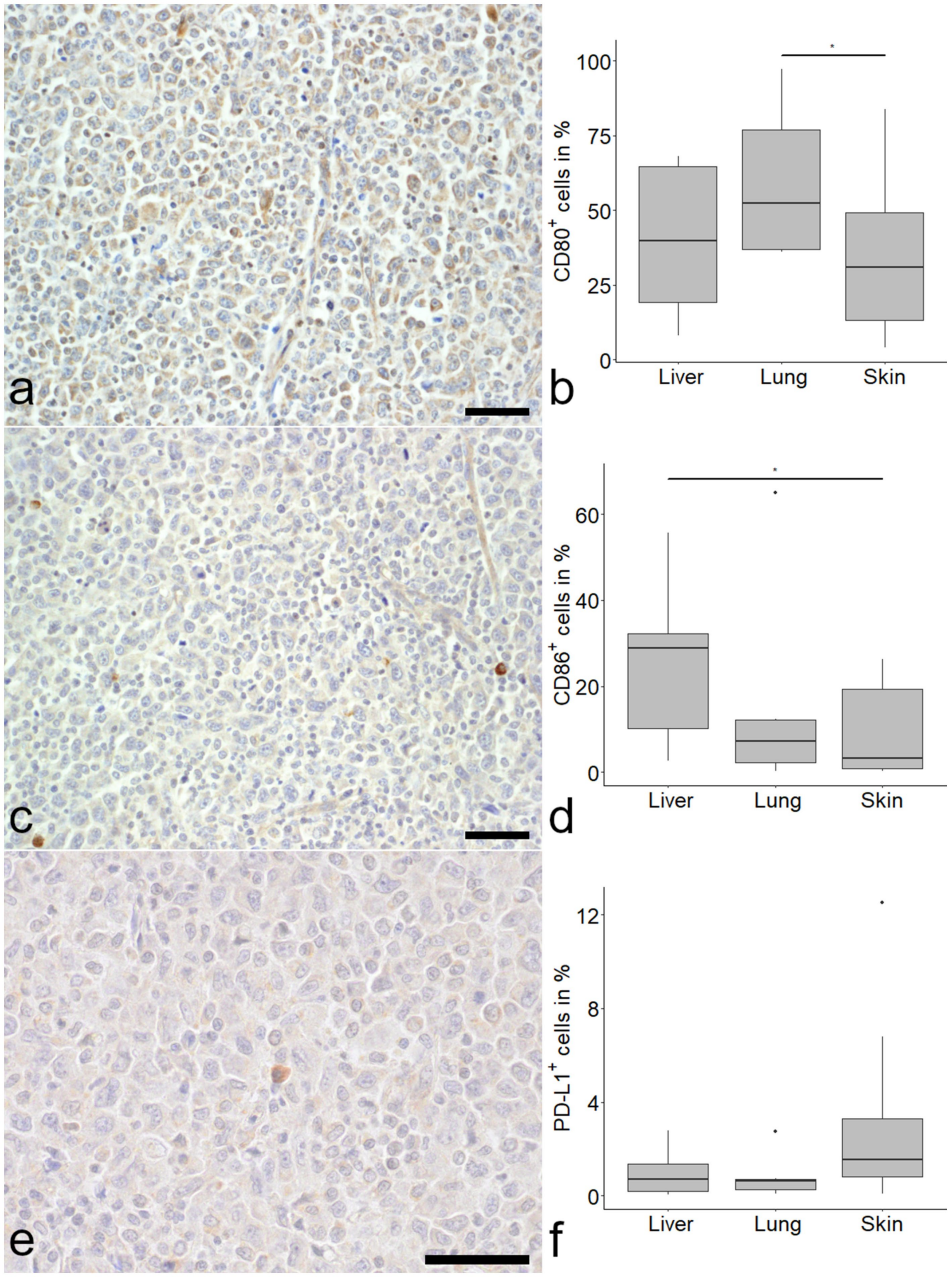
Expression of immune checkpoint molecules in histiocytic sarcoma (HS). CD80 was frequently detected in tumor cells **(a)**. CD80 positive tumor cells were more frequent in the lung compared to the skin **(b**; Kruskal–Wallis-test with Wilcoxon-rank-sum-post-hoc test, *p* < 0.05). The number of CD86 immunolabeled cells was markedly lower in skin **(c)** and lung, with a significant difference between liver and skin **(d)**. Programmed death ligand 1 (PD-L1) was expressed in a minor proportion of cells **(e)** with no difference between organs **(f)**. Mayer’s hematoxylin counterstain, bar = 50 μm.

CD80 was detected in all samples with the highest proportion of positive cells in the lung (Figure 2). Median CD80 expression levels were 39.78, 52.29 and 30.81% in liver, lung and skin respectively, with a difference between lung and skin (Figure 2).

CD86 expression was most pronounced in the liver with a median of 28.77%, compared to 7.21 and 3.19% in lung and skin, respectively. A difference was seen between liver and skin (Figure 2).

PD-L1 expression was relatively low in all samples, with a median percentage of positive cells of 0.70% in the liver, 0.61% in the lung and 1.54% in the skin (Figure 2).

Throughout the organs a positive correlation of CD86 and Casp-3 was detected (*ρ* = 0.58, *p* < 0.05). This correlation was even stronger if lung samples were investigated separately (*ρ* = 0.83, *p* < 0.05). No correlation was found between PD-L1 and Casp-3 or Ki-67 when all organs were examined together. However, a negative correlation for Ki-67 and PD-L1 in the lung (*ρ* = −0.83, *p* < 0.05) and a positive correlation (*ρ* = 0.62, *p* = 0.05) for PD-L1 and Casp-3 in the skin were detected. Interestingly, CD80 showed a positive correlation with survivin (*ρ* = 0.44, *p* < 0.05).

**Figure 3:**
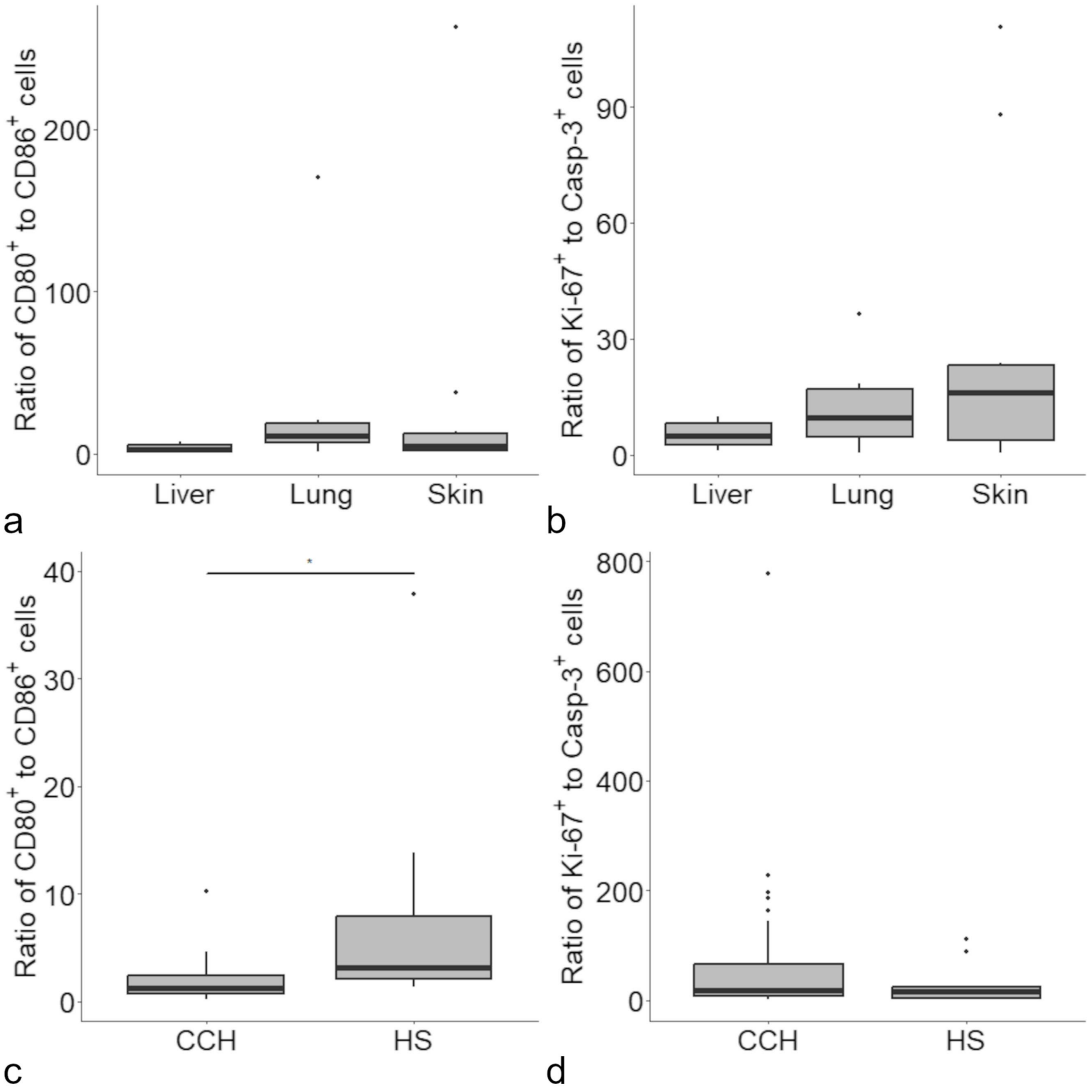
Ratios were calculated for the proportion of CD80 and CD86 immunolabeled cells in histiocytic sarcoma (HS), with no difference between organs **(a)**. A similar approach revealed no difference between the ratio for Ki-67 and cleaved caspase-3 (Casp-3) immunolabeled cells between the investigated organs **(b)**. Upon comparison of the present data with data obtained from canine cutaneous histiocytoma [CCH (11)] a significant difference was detected for the ratio of CD80/CD86 positive cells **(c)** but not for Ki-67/Casp-3 positive cells **(d)**.

The ratio of CD80 and CD86 positive cells was calculated to identify a potential role of CD80 dominance. Median ratios were 2.33 for liver, 10.85 for lung and 4.48 for skin (Figure 3). No statistically significant difference was found between organs. A moderate negative correlation of the ratio with Casp-3 was found in lung and skin (pooled correlation values: *ρ* = − 0.69, *p* < 0.05).

A similar ratio was calculated for Ki-67 and Casp-3 and used as a presumptive measure of “tumor growth” (Figure 3). Correlations of the Ki-67/Casp-3 ratio with neither CD80, CD86 nor survivin did reach the level of significance.

The calculated ratios were also used in relation to our previous study on CCH (11). A difference was found for the CD80/CD86 ratio of CCH in comparison with (sub) cutaneous HS (medians of 1.31 and 4.48, respectively; Figure 3). Further, a proliferation ratio was calculated in the same manner for proportion of Ki-67/Casp-3 positive cells. Here, no significant difference was found for CCH and HS (medians of 18.72 and 16.10, respectively; Figure 3).

### 3.4 IFN-pathway markers

**Figure 4:**
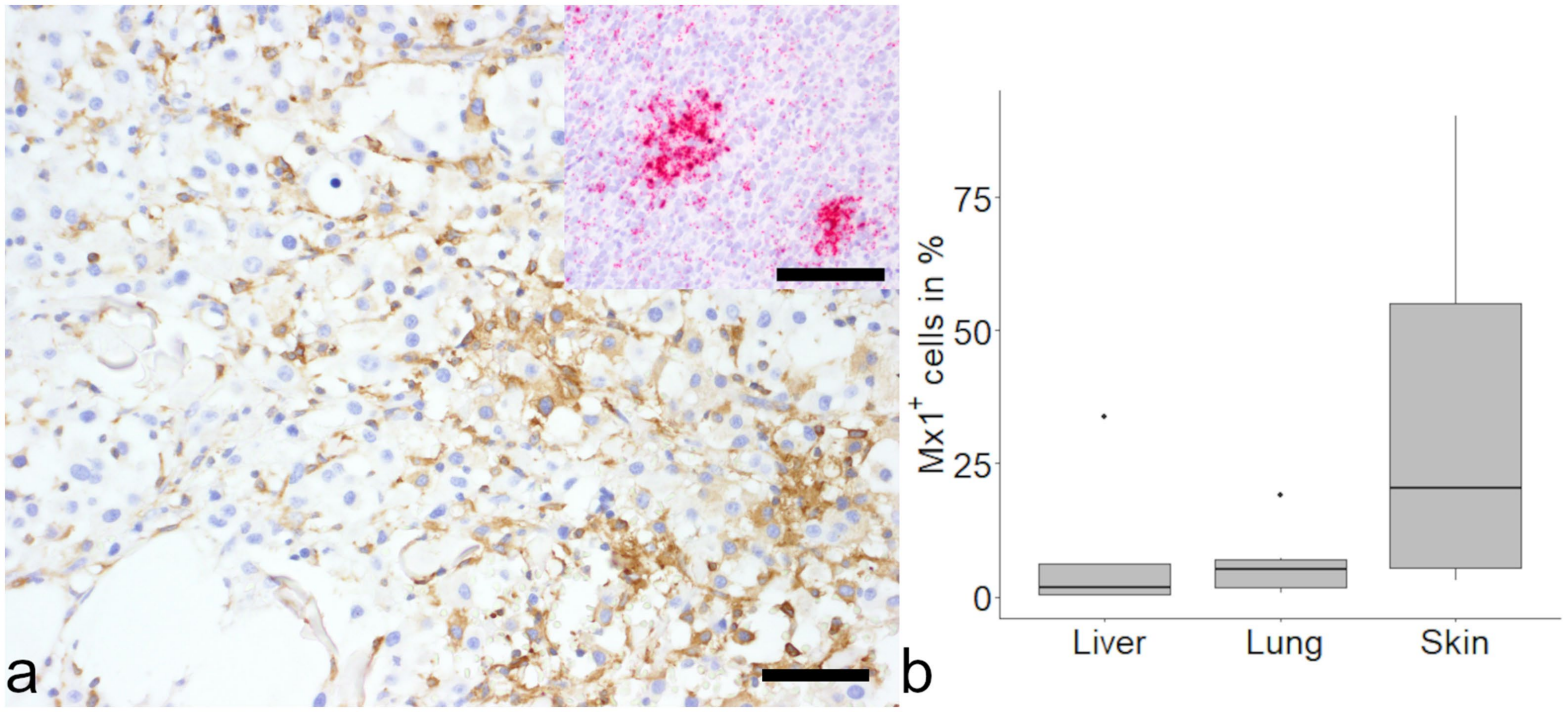
Evaluation of MX dynamin like GTPase-1 (mx1) in histiocytic sarcoma (HS). Positive signal was found in samples from cutaneous histiocytic sarcoma via immunohistochemistry (**a**, large image) and *in-situ* hybridization (**a**, inset). No differences were found between organs **(b)**. Mayer’s hematoxylin counterstain, bar = 50 μm.

Over 50% of HS cells in the skin showed detection of mRNA transcripts of mx1 and 20% of cells in the skin were immunopositive for mx1 (Figure 4). Interestingly, in liver and lung a small number of cells showed mx1 expression (1.60 and 5.25% respectively) but the differences did not reach the level of significance.

In addition, more than 75% of cells in skin and liver were immunopositive for STING, while 35% of cells in the lung were identified as positive.

## 4 Discussion

The aims of this study were to verify an effect of immune checkpoint molecules in HS, to elucidate possible differences in affected organs and to determine a role of mx1 as marker of the IFN-I-pathway in HS.

In the present study, an expression of CD86 in tumor cells was accompanied by an increased number of Casp-3 expressing cells in HS in lung and skin. Additionally, the correlation of CD80 with survivin points to the presence of a direct or indirect anti-apoptotic effect. However, the exact mechanism remains elusive. CTLA-4 can prevent binding of CD80 and CD86 to CD28 because of a greater affinity to these molecules leading to an inhibitory effect (34). Interaction of CTLA-4 and CD80 leads to the degradation of both molecules resulting in the attenuation of T cell effector mechanisms (34). In contrast, if CD80 and CD86 are co-expressed, CD86 may trigger a co-stimulatory effect by binding to CD28 (34, 35). The results of the present study are consistent with the mechanisms described in the literature and suggest an anti-tumor effect of CD86 mediated by immune cells.

In the present study, an analysis of skin, lung and liver samples revealed differences in the expression of CD80 and CD86. Notably, a study comparing soft-tissue and splenic HS identified differences in the number of regulatory T-cells and naïve T-cells in Flat Coated Retrievers (36). Therefore, the aforementioned differences may be attributed to the influence of organ-specific immune and stromal cells as well as the extracellular matrix (37).

In contrast to skin and lung, the liver is not described as primary localization of HS. It seems feasible that the comparable level of CD80 and CD86 found in the liver but not in the other organs, could point to an effect due to the metastatic origin. Differences in the TME in primary and metastatic locations have been described in other neoplasia and are partly attributed to a priming by the neoplastic cells, often resulting in a decreased response to therapy in metastasis (37, 38). No studies have investigated these relations in canine neoplasia.

In CCH, CD80 showed a declining expression, which is putatively related to tumor regression, while CD86 showed comparable levels (11). Thus, a relative increase in CD86 may be related to an anti-TME: the distinct ratios of CD80 and CD86 in CCH and HS suggest an involvement of these molecules in the behavior of the investigated neoplasia. In addition, a similar difference between CCH and HS regarding CD80 and CD86 on mRNA level was recently described (12).

It should be considered that most skin samples were collected by excisional biopsy and may present localized HS, while internal organs were collected from necropsy cases with disseminated HS. The lack of statistical significance comparing expression levels for CD80 and CD86 between liver and lung is likely due to the low sample size.

Despite the small proportion of PD-L1 positive cells, the data suggest a negative relation with mitosis and a positive relation with apoptosis in canine HS. In contrast to our study, Hartley et al. (39) showed a high PD-L1 expression on frozen-sectioned material from canine HS using immunohistochemistry in one dog and Murphy et al. (40) used *in-situ*-hybridization and detected high levels of PD-L1 mRNA in neoplastic cells from canine HS. Another study using immunohistochemistry on canine FFPE of 5 HS found no expression of PD-L1 (41). Differences may be attributed to the techniques or sample size used and low values may even be attributed to resident histiocytes. Pinpointing a threshold without clinical follow-up is hard as for PD-L1 even 1% have been shown as relevant for an effect on patient survival (42). In the present study, due to a lack of follow-up data, a clinical effect of PD-L1 cannot be determined, but another study evaluating PD-L1 mRNA detections in HS found no influence on survival (40).

Interestingly, high levels of PD-L1 were detected in CCH (11). This may be related to an increase of PD-L1 in the presence of IFN-γ, as seen in CCH but not in HS (39).

Monoclonal antibodies targeting the immune checkpoint receptors PD-1 and CTLA-4 are implemented in the treatment of human cancer (43). In dogs, studies show limited efficacy of a PD-1 antibody (44). No antibodies targeting CTLA-4 have been approved for canines yet, but *in vitro*-studies show their principal functionality (45). Thus, the findings of this study indicate that dogs suffering from HS may benefit from treatment with monoclonal antibodies targeting immune checkpoint receptors.

Considering effects of IFN in HS, the upregulation of mx1 previously described in CCH (11) was also detected in (sub) cutaneous HS. The additional detection of STING in HS points to an activated IFN-I-pathway in this neoplasia. In this context, mx1 has been described as part of a subset of IFN-stimulated genes expressed due to a chronic IFN-I stimulation with a beneficial role for the tumor (26). In HS, a pro-tumor effect seems likely due to the progressive nature of this neoplasm.

The heterogenous and high expression of survivin found in HS could be linked to its biological behavior. In cell culture of canine HS cells, survivin has been linked to an aggressive cellular behavior (46). In the present study, intranuclear survivin expression correlates with Ki-67 expression in HS. This result is supported by a study showing a co-localization of nuclear survivin and Ki-67 (47) and in line with its role in mitosis (48). Nevertheless, the intracytoplasmic localization is often seen in cancer (49). A dominance of intracytoplasmic survivin was related to a pro-mitotic function in CCH (11). In conclusion, evaluating the subcellular localization of survivin might benefit studies investigating survivin in canine neoplasia. For example, in human cancer studies nuclear survivin localization is often associated with a worse prognosis (50). In animals, further studies are needed to link clinical behavior to survivin expression and investigate a possible therapeutic potential of survivin inhibitors on HS *in vivo*.

## 5 Conclusion

In the present study, a link of an increased number of CD86 positive cells to pro-apoptotic events seems likely, similar to the effect of CD80 and CD86 described in CCH. The evaluation of the ratio of CD80 and CD86 may serve as a tool for future research in canine neoplasia. The organ specific differences detected in this study point to the importance of an organ-specific TME in canine HS. The link of PD-L1 to mitotic and apoptotic markers raises questions about the correlation of positive expression and biological influence. As previously described in CCH an expression of mx1 was found in HS indicating a tumor-beneficial role of a subset of IFN-stimulated genes in canine HS.

## Data Availability

The raw data supporting the conclusions of this article will be made available by the authors, without undue reservation.
